# A randomized, double-blind, placebo-controlled trial of vitamin D supplementation with or without calcium in community-dwelling vitamin D deficient subjects

**DOI:** 10.1186/s12891-022-05364-z

**Published:** 2022-05-03

**Authors:** Salah Gariballa, Javed Yasin, Awad Alessa

**Affiliations:** Internal Medicine, Faculty of Medicine & Health Sciences, United Arab Emirates University, PO Box 17666, Al Ain, United Arab Emirates

**Keywords:** Vitamin D, Calcium, Placebo, Bone turnover, Inflammation, Body pains, Sun exposure

## Abstract

**Background:**

Although vitamin D deficiency is highly prevalent in the Middle East, very few studies have attempted to measure its health impact.

**Aims:**

We aimed to assess whether vitamin D3 and calcium, either alone or in combination, have health benefit.

**Methods:**

In a 2 × 2 factorial design double-blind, placebo-controlled trial, Community free living adults living in the city of Al Ain, UAE were randomly assigned to receive daily 2000 IU oral vitamin D3 alone, 600 mg calcium alone, oral vitamin D3 (2000 IU per day) combined with 600 mg calcium, or a placebo for 6 months. Primary outcomes were self-rated health and bone turnover markers.

**Results:**

Of the 545 randomized, 277 subjects completed 6 months follow up. 25(OH)D levels marginally increased in the two groups received vitamin D3 alone or combined with calcium compared to the decline seen in those who received calcium supplement alone or a placebo. Sub-group analysis revealed that parathyroid hormone (PTH) concentration decreased and Calcium/creatinine ratio increased significantly in the combined vitamin D and Calcium group compared to the vitamin D alone or Calcium alone in contrast to the increase seen in the placebo group [*p* < 0.05 for between group difference at 6 months]. There were no statistically significant differences between the supplement and placebo groups at the 6 months follow-up in body weight, body mass index (BMI), blood pressure, body pains and general health.

**Conclusion:**

PTH concentration decreased and calcium/creatinine ratio increased in subjects who received vitamin D and Calcium together compared to those who received vitamin D alone.

**Trial registration:**

NCT02662491, First registered on 25 January 2016 (https://register.clinicaltrials.gov/prs/app/action/SelectProtocol?sid=S00060CE&selectaction=Edit&uid=U0001M6P&ts=3&cx=scu4cb, Last update: 05 August 2019.

## Background

Vitamin D an active hormone is essential for both bone and muscle health because of it is role in calcium and phosphorus homeostasis [1]. Evidence also points to other health benefits of vitamin D because most cells in the human body have vitamin D receptors [1]. This is strengthened further by the results of some observational studies which have reported increased risks of cardiovascular, malignant, and autoimmune diseases, however many recent studies did not confirm these results [1].

Sunlight, diet, and dietary supplements are the main sources of vitamin D for humans. Other factors which may also affect vitamin D status includes skin pigmentation, age, smoking and adiposity [1, 2]. Low circulating vitamin D levels [25(OH)D] have recently been associated with increased obesity and associated cardiometabolic risk factors and pathologies including diabetes. In the United Arab Emirates (UAE) we have the 2nd highest rates of obesity in the World. In addition, several studies have revealed that vitamin D deficiency is highly prevalent in the UAE, Middle East and the Indian subcontinent [3, 4]. For example, a recent study reported that vitamin D deficiency is reaching epidemic proportions in the Indian subcontinent, with a prevalence of 70–100% in the general population [4]. Another recent study from the UAE reported the prevalence of vitamin D deficiency to be around 83.1% in male subjects and 83.8% in female subjects [3]. However most of these studies so far were either of small size and/or cross-sectional nature. In addition, some of the causes and more importantly the health consequences of low vitamin D levels are not currently known. For example, some studies on the impact of vitamin D deficiency on bone health in some Asian populations reported conflicting results [5–7]. Altered metabolism of vitamin D in Asian women with vitamin D deficiency may be one of the reasons which protect them from bone loss. Another explanation is that genetic and/or ethnic difference may influence vitamin D metabolism in these populations [6, 8]. This is clearly an area for research. Emerging evidence is also suggesting that increasing or adequate calcium intake by diet or use of supplement may mitigate the deleterious effects of low vitamin D concentrations on bone health by preventing or reducing bone turnover especially in darker-skinned people [7–9]. The minimum amount of dietary calcium required to induce this response is also not clear and may vary with ethnicity [7]. Again, very few studies have attempted to measure calcium intake in relation to bone health in areas where vitamin D deficiency is prevalent [7, 9–11]. Research is also required to examine the threshold of vitamin D and calcium intake and/or level in relation to bone turnover and other health outcomes in the UAE population. Although there is some evidence that vitamin D deficiency is highly prevalent in UAE citizens, very few studies have attempted to measure its health implications and potential clinical benefits of optimizing vitamin D and/or calcium status in the UAE population. The aim of this trial was to examine whether vitamin D3 and calcium supplementation, either alone or in combination, have health benefit.

## Methods and study design

Apparently healthy community free-living Emirati (UAE citizens) and expatriates, Arabian men & women aged 18 years and over were recruited for this randomized, double-blind placebo-controlled placebo intervention trial from January 2017 to December 2019. Subjects were recruited by advertisement through the local press, from community health centers and from hospital out-patient in Al Ain city which has 2 main teaching hospitals serving a total population of 400,000. Individuals with renal disease or stones, parathyroid disease, hypercalcemia, on calcium and/or vitamin D supplementation, bisphosphonates, steroid medications, hormones or diuretics or unable to give an informed written consent were excluded. Al Ain Medical District Human research ethics committee approved the study protocol and consent to participate and all participants provided informed written consent.

### Study design

The study was a two by two factorial, randomized controlled intervention trial. Participants were allocated to 4 equal groups and assigned one tablet once daily containing 2000 IU of vitamin D3, 600 mg of calcium, vitamin D3 (2000 IU) combined with 600 mg calcium, placebo for 6 months. Tablets in all 4 groups were of equal size and had an identical appearance. Participants, research nurse and all investigators were blinded to the intervention assignment throughout the trial. Compliance was assessed by counting tablets at follow up appointments. Tablets were given to participants and instructed to take them for 6 months. Randomization was done by generation of a random sequence achieved using a computer random number table. Following informed written consent of eligible subject’s blood and urine samples were taken for measurements of 25(OH)D, markers of bone turnover and related biochemical variables. Clinical assessment that included general and self-rated health, body pains, physical activity and dietary intakes performed at baseline, and repeated at 6 months post-randomization. Outcome measures were biochemical variables of metabolic risk factors, bone turnover (biochemical measures of bone metabolism) and muscle and general health. The dietary supplement tablets ceased after collection of the 6-month blood & urine samples. Participants were not given advise related to dietary vitamin D and or calcium intake. Information on other important variables likely to influence vitamin D status including age, reproductive history, smoking, medications, adiposity, exposure to sunlight, dietary intake including supplements, chronic illness and medications were collected and adjusted for during the analysis.

#### Role of supplements manufacturer and funding body

Supplements and placebo were purchased commercially (supplied by the Nutraceutical and Vitamin Supplement Manufacturing company ECKHART(ECKHARTcorp.com), Novato, CA 94945 USA). The UAEU funded the research. The funding body and the supplier played no part in the trial design or in the collection, analysis, or interpretation of data.

#### Measurements

Details of the methods including some of the clinical and biochemical measurement were published before [12]. Briefly face-to-face qquestionnaire data were collected on lifestyle and health factors that are of interest in this study of the health implications of vitamin D deficiency in UAE citizens. A common set of questions on education and socio-economic status; current and past occupation; history of previous illness or surgical operations; tobacco smoking; consumption of beverages; physical activity; reproductive history; use of herbal medicine, vitamin supplements, exogenous hormones for contraception and postmenopausal replacement therapy, sun exposure and body pains. Diet: Intake of calcium and vitamin D was assessed by food-frequency questionnaire developed and validated by the vitamin D research group [13]. A validated questionnaire was used to assess occupation and leisure-related physical activity [14]. Data were obtained on frequency and duration of daily or weekly physical activity sessions for at least 20 minutes or more in which subjects became breathless or sweating. Sunlight exposure was assessed by a questionnaire about dress, time spent outdoor during different seasons and use of protective sun screen including creams. Height, weight and blood pressure were also measured using standard methods.

#### Biochemical and urine analysis [12]

Patients provided a fasting morning blood sample after which a spot urine sample was also obtained. Biochemical analyses included 25(OH)D, Total P1NP (total Procollagen type 1 amino-terminal propeptide), Osteocalcin (OCN) and PTH (Parathyroid Hormone) were measured using fully automated COBAS e411 analyzer that uses a patented Electro Chemiluminescence (ECL) technology for immunoassay analysis), from ROCHE diagnostics, Manheim; U PYD = Human Pyridinoline (PYD) measured using ELISA Kit from MyBioSource, USA Cat. No. MBS030461; U DPD = Human Deoxypyridinoline (DPD) measured using ELISA Kit from MyBioSource, USA Cat. No. MBS039364; CTX-1 = Human cross-linked carboxy-terminal telopeptide of type I collagen measured using ELISA Kit Cat. No. MBS700254. Other biochemical variables including routine baseline tests including lipid profile (Cholesterol, Triglyceride, HDL, LDL), HbA1c, Blood Glucose, high sensitivity C-reactive protein (Hs-CRP), Calcium, Phosphorus, Urine- Creatinine were measured using standard protocols already established at our two teaching hospitals.

#### Sample size calculation

Sample size was based on a factorial design**.** Saadi et al. have previously shown that supplementation of nulliparous women with 2000 IU vitamin D_2_ for 3 months diet increased 25(OH)D from 21.0 ± 12.3 to 40.2 ± 23.0 nmol/L and decreased clinical symptoms of muscle cramps (66% compared with 4%, *p* < 0,05) [15]. We propose therefore that after 6 months of intervention a sample of 332 (83 subjects in each one of 4 groups) would allow the detection of 20% reduction in clinical symptoms and significantly increase 25(OH)D levels. Taking into-account an expected drop-out of 30%, recruitment of 432 subjects (108 for each group) will give us 90% power to detect a difference in clinical symptoms and 25(OH)D levels of this magnitude at the α level of 0.05. The achieved sample size of 277 (68 subjects received vitamin D, 68 vitamin & calcium, 75 calcium and 66 controls) would allow the detection of a true mean difference of 2.3 nmol/L in 25(OH)D with a standard deviation of 5 nmol/L between the intervention and control group at the α level of 0.05 with probability (power) 0.9. This sample would also allow the detection of 22% reduction in clinical symptoms.

### Data analysis

All statistical analyses were based on an intention-to-treat basis for those subjects who completed 6 months follow up with a *p*-value of < 0.05 regarded as statistically significant. All statistical analyses were done with blinding maintained. The code was broken only after data collection had been completed. The primary time point of interest was 6 months post randomization. Repeated-measures analysis of variance used to assess between group difference in cumulative changes at 6 months. All data analyzed using the SPSS statistical package.

## Results

**Fig. 1:**
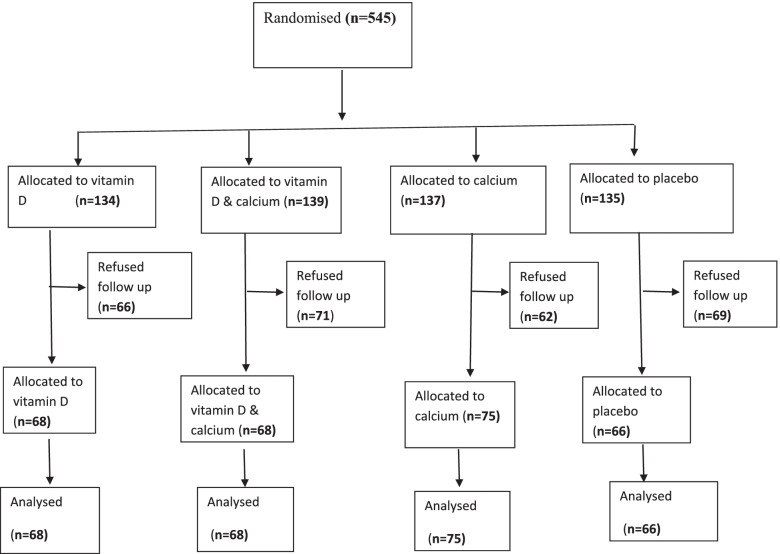
Enrolment, treatment and 6-month follow up of study subjects

Figure 1 shows the trial profile. Of the 545 subjects randomized, 277 participants completed 6 months follow up. There were no statistically differences in baseline characteristics including sex, smoking, chronic diseases, medications and physical activities between those who completed 6 months follow up (responders = 277) and those who did not come for follow up (non-responders = 268) except for age [responders age = 41 years (SD 11) compared with 37 years [11]] in non-responders (*p* < 0.01). The main reasons for exclusion were non-response to repeated attempts to provide follow up outcome data including biological samples.

Baseline demographic and clinical characteristics of responders including physical activity, general health and body pains, were well balanced across the groups except for prevalence of diabetes (Table 1). No significant differences were found in calcium and vitamin D dietary intakes between the supplement and control groups.

**Table 1:** Baseline characteristics at trial entry by randomization group, mean (SD) unless stated otherwise

	Vitamin D3 (***n*** = 68)	Vitamin D3 + Calcium (***n*** = 68)	Calcium (***n*** = 75)	Placebo (***n*** = 66)	*P* value
**Age (years)**	42 (11)	41 (13)	41 (13)	41 (13)	0.89
**Gender, n (%)**					
female	55 (78)	55 (73)	48 (73)	48 (70)	0.28
**Smoking, n (%)**					0.945
Yes	8 (11)	10 (13)	7 (11)	10 (15)	
Occasionally	5 (7)	3 (4)	2 (3)	2 (3)	
No	55 (79)	63 (82)	55 (83)	55 (80)	
**Diabetes mellitus, n (%)**^**a**^	7 (10)	10 (13)	12 (18)	17 (25)	0.02
**Hypertension, n (%)**^**a**^	8 (11)	11 (15)	9 (14)	14 (20)	0.14
**Body mass index (BMI)**	29.3 (5)	28.7 (5.5)	29 (6)	28.6 (5)	0.99
**Systolic blood pressure**	118 (8)	120 (9)	116 (10)	122 (14)	0.35
**Diastolic blood pressure**	75 (6)	79 (5)	76 (6)	77 (6)	0.28
**Cholesterol (mmol/L)**	7 (10)	8 (11)	4 (6)	14 (20)	0.08
**Calcium (mmol/L)**	2.28 (0.1)	2.29 (0.1)	2.27 (0.1)	2.27 (0.09)	0.98
**Vitamin D (ng/ml)**	22.8 (9)	19.0 (11)	25.4 (11)	25.1 (11)	0.58
**Physical activity n (%)**					0.65
Very active	18 (25)	17 (23)	12 (18)	8 (12)	
Moderate	41 (59)	47 (62)	37 (57)	49 (72)	
Not active	11 (16)	17 (22)	16 (25)	11 (16)	
**Body pains, n (%)**					0.97
yes	27 (38)	23 (31)	21 (32)	26 (38)	
no	43 (62)	52 (69)	44 (67)	42 (62)	
**General Health, n (%)**					0.99
excellent	6 (8)	9 (12)	6 (9)	4 (6)	
good	58 (78)	55 (71)	52 (76)	54 (76)	
fair	10 (14)	9 (12)	8 (12)	8 (11)	
poor	0 (0)	4 (5)	2 (3)	5 (7)	

^**a**^history of previously diagnosed diabetes mellitus or hypertension

**Fig. 2:**
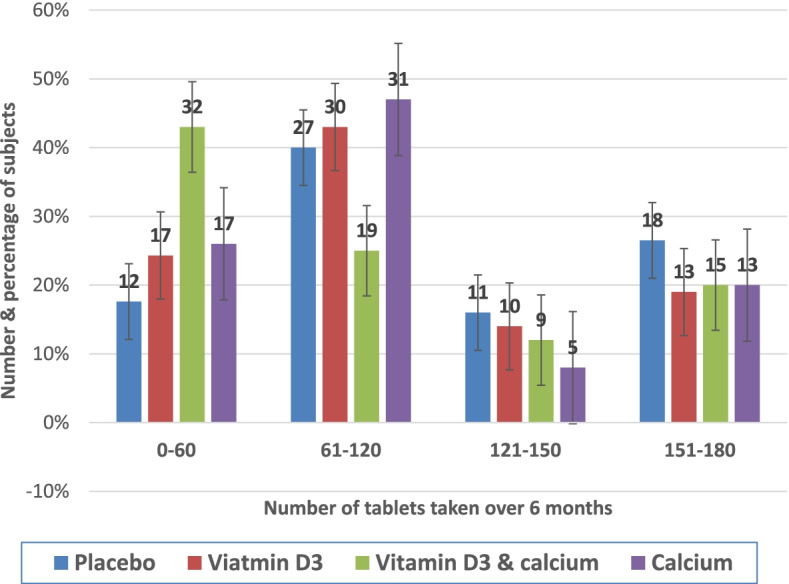
Compliance with supplement and placebo (maximum 180 tablets)

Figure 2 shows compliance with trail supplement and placebo tablets expressed as subjects consuming tablets, divided into 4 quartiles. Although 25(OH)D levels marginally increased in the two groups received vitamin D3 supplement compared to the decline seen in the calcium supplement alone and the placebo group, these changes between groups albeit more pronounced in subjects who took 50% or more of prescribed supplements tables, did not reach statistical significance.

**Fig. 3:**
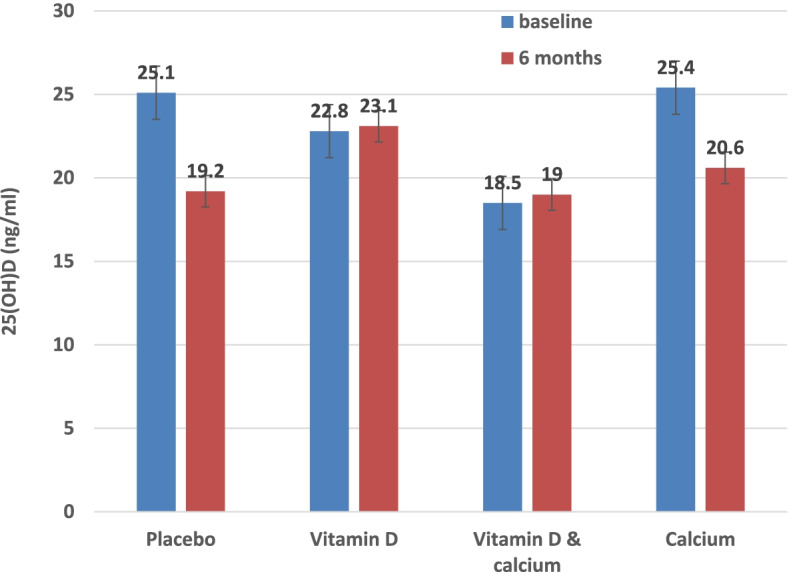
Effect of vitamin D3 alone, Calcium alone or in combination compared to placebo on 25(OH)D concentrations at 6 months follow up. *P* value = 0.12, for between group difference in cumulative changes at 6 months. Results adjusted for BMI, sun exposure, diet & physical activity

The effect of intervention on vitamin D, metabolic, inflammatory and bone turnover markers is shown in Table 2. With the exception of PTH and Ca/Cr, there were no statistically significant differences between the supplement and placebo groups at the 6 months follow-up. Although 25(OH)D levels marginally increased in the two groups received vitamin D3 supplement compared to the decline seen in the calcium supplement alone and the placebo group, these changes between groups albeit more pronounced in subjects who took 50% or more of prescribed supplements tables, did not reach statistical significance (Fig. 3). Gender, age but not body mass index, sun exposure, diet or physical activity influenced response to supplements but the effect is statistically significant for gender only. In subjects who received 120 (50th quartile) or more supplement tables, PTH concentration decreased significantly in the combined vitamin D and Calcium group compared to the vitamin D alone or Calcium alone in contrast to the increase seen in the placebo group [*p* < 0.05 for between group difference at 6 months.

**Table 2:** Main biochemical & bone turnover outcomes by randomized group, mean (SD) unless stated otherwise

Clinical variable	Vitamin D3 (***n*** = 68)		Vitamin D3 + Calcium (***n*** = 68)		Calcium (***n*** = 75)		Placebo (***n*** = 66)	
	Baseline	6 months	Baseline	6 months	Baseline	6 months	Baseline	6 months
HbA1C (%)	5.85 (0.9)	5.82 (0.98)	5.96 (0.99)	5.75 (1.4)	6.08 (1.1)	5.86 (1.0)	6.1 (1.3)	6.02 (1.4)
Hs CRP (mg/L)	2.91 (2.8)	3.48 (3.9)	3.1 (3.5)	3.3 (4)	4.6 (6)	4.03 (4)	3.3 (3.3)	3.06 (3.7)
Calcium (mmol/L)	2.29 (0.1)	2.16 (0.2)	2.29 (0.1)	2.18 (0.2)	2.25 (o.1)	2.16 (0.2)	2.28 (0.1)	2.18 (0.2)
PTH (pg/ml)^*^	30 (17)	29 (21)	29 (11)	22 (8)	24 (12)	22 (9)	22 (13)	24 (10)
Osteocalcin (ng/ml)	17 (6)	22 (10)	17.8 (6.5)	19 (6.8)	16 (10)	19 (11)	16 (7.4)	19.6 (8)
P1NP (ng/ml)	50 (21)	48 (25)	50 (22)	43 (19)	45 (30)	47 (27)	46 (27)	45 (21)
CTX1 (ng/ml)	10 (6)	7.3 (4.7)	10 (7.6)	8.4 (6)	10.5 (7)	7.6 (5)	12.5 (8)	8.8 (6)
U-DPD (nmol/L)	102 (49)	98 (46)	91 (46)	83 (41)	79 (42)	74 (41)	100 (47)	92 (49)
U-PYD (nmol/L)	217 (97)	239 (126)	247 (73)	284 (116)	196 (86)	241 (126)	242 (121)	236 (115)
PYD/CR	20.8 (11)	22 (11)	24.7 (11)	30.2 (12)	24.9 (11)	30 (18)	23.7 (10)	22.7 (8)
DPD/CR	9.2 (4)	9.0 (4.4)	9.5 (5.6)	9.1 (4.4)	10.7 (6.5)	9.7 (6)	10.1 (4.5)	9.1 (4)
Ca/Cr^*^	0.201 (0.06)	0.206 (0.06)	0.208 (0.13)	0.255 (0.09)	0.313 (0.13)	0.306 (0.014)	0.204 (0.08)	0.215 (0.09)

*Abbreviations*: *HsCRP* High sensitivity C reactive protein, *PTH* Parathyroid Hormone, *P1NP* Procollagen type-1 N-terminal propeptide, *CTX1* C-terminal telopeptide of type −1 collagen, *U DPD* Urine Deoxypyridinoline, *U PYD* Urine Pyridinoloine, *OSTEO* Osteocalcins, *PYD/Cr* Human Pyridinoline/creatinine, *DPD/Cr* Human Deoxypyridinoline/creatinine, *Ca/cr* Calcium/creatinine

^*^p value < 0.05 for between group difference in cumulative changes at 6 month

There were no statistically significant differences between the supplement and placebo groups at the 6 months follow-up in body weight, BMI, blood pressure, body pains and general health (Table 3). No other clinically important difference between supplement and placebo group was found in physical activity, dietary intakes, sun exposure, infections or supplements-related side effects (results not shown).

**Table 3:** Main clinical outcomes by randomized groups, mean (SD) unless stated otherwise

Clinical variable	Vitamin D3 (***n*** = 68)		Vitamin D3 + Calcium (***n*** = 68)		Calcium (***n*** = 75)		Placebo (***n*** = 66)	
	Baseline	6 months	Baseline	6 months	Baseline	6 months	Baseline	6 months
Body weight (kg)	79(15)	79.5(15)	79.2(17)	79.9(17)	79.6(16)	79.9(17)	79.3 (16)	79.8 (16)
BMI	29.3 (4.8)	29.6 (4.7)	28.7(5.5)	29(6)	29(6)	29.2(6)	28.6 (5)	28.7 (5)
SBP (mmHg)	118(8)	120(6)	120(9)	122(9)	116 (10)	120(8)	122 (14)	123 (9)
DBP (mmHg)	75(6)	76(6)	79(5)	78(6)	76(6)	77(7)	77 (6)	81 (12)
Body pains, n (%)								
Yes	27(38)	16(23)	23(31)	21(28)	21(32)	13(20)	26 (38)	15 (29)
No	43(61)	45(64)	52(69)	41(66)	44(67)	38(58)	42 (62)	37 (71)
Pain interferes with sleep, n (%)								
Yes	19(27)	11(16)	9(12)	6(8)	7(11)	2(3)	13 (19)	5 (7)
No	51(73)	50(71)	66(88)	50(67)	58(88)	46(70)	55 (81)	46 (68)
Pain interferes with daily activities**,** n (%)								
Yes	16(23)	10(14)	10(13)	7(9)	5(8)	5(8)	13 (19)	6 (9)
No	54(77)	51(73)	65(87)	50(67)	60(91)	43(65)	55 (81)	43 (63)
General Health, n (%)								
Excellent	13(19)	6(9)	15(20)	9 (12)	10(15)	6(9)	9 (13)	3 (4)
Good	38(54)	53(77)	39(52)	53(70)	42(64)	50(76)	45 (66)	51 (76)
Fair	12(17)	10(14)	15(20)	9(12)	7(10)	8(12)	4 (6)	8 (12)
poor	4(6)	0(0)	5(7)	4(5)	4(6)	2(3)	8 (12)	5 (8)

^*^p value < 0.05 for between group difference in cumulative changes at 6 months

## Discussion

In this randomized, double-blind, placebo-controlled trial in community-dwelling vitamin D deficient UAE citizens, we found no evidence of a clinical benefit of vitamin D supplementation with or without calcium. We did however find decreased PTH concentration and increased calcium/creatinine ratio in subjects who received vitamin D and Calcium together compared to the vitamin D alone, calcium alone or the placebo group. The current study is, to our knowledge, the first randomized, double-blind, placebo-controlled trial to investigate the effect of vitamin D supplementation with or without calcium on metabolic and bone turnover markers and general health in community-dwelling vitamin D deficient UAE citizens.

Several possible reasons may explain the lack of potential benefits of the supplements in our study. First, the large number of randomized subjects who did not come for the 6-month follow up. However, baseline demographic and clinical characteristics were well balanced across the groups who had follow data compared to those who did not come for the follow up. In Addition, and based on pre-study sample size calculation the number of participants with follow up data is large enough to detect a difference if one exists. Second, 25(OH)D levels did not show the expected increase in relation to supplements dose and duration albeit this was slightly better in subjects who reported taking more than 120 (50th quartile) of prescribed trial medications. This could be a true finding or alternatively the result of over reporting of the number of tables taken although compliance was assessed meticulously by counting tablets at follow up appointments in all study participants. Another reason which could explain the variability in response to supplements is the high proportion of overweight and obese subjects [198 (70%)] in our study population because overweight and obesity were reported to markedly decrease response to vitamin D supplementation [16, 17]. Recent research evidence including some health professional bodies recommend that obese subjects be given two to three times more vitamin D doses compared to normal weight subjects and depending on 25(OH)D target level [18]. Although there is no consensus as to the reasons for the poor vitamin D status and response to supplements in obese subjects a number of studies have reported different mechanisms including decrease absorption, greater volume of distribution and tightly bound vitamin D in fatty tissues [16, 18–20]. Another possible reason for the low circulating levels of vitamin D in obese subjects are diets high in carbohydrates and low vitamin D [21]. Indeed, our society in the UAE has been through rapid socioeconomic and social changes with urbanization over the last 40 years. Accompanying changes in diet and lifestyle are leading to growing epidemic of overweight/obesity, diabetes and other related conditions including vitamin D deficiency. However, adjustment for BMI, physical activity, sun exposure and vitamin D & calcium rich diet (result not shown) did not show significant association with baseline vitamin D levels or response to supplements. Recently vitamin D supplementation to reach and sustain 25(OH)D levels of 100–124 and ≥ 125 nmol/l has been shown to convey a progressively lower risk of progression to diabetes in adults with prediabetes [22]. In the light of recently accumulating evidence, there an urgent need however, to study the effects of higher dose, frequency and duration of vitamin D supplementation to reach and sustain higher levels of 25(OH)D to find out if indeed vitamin D supplementation have clinical benefits in our high-risk population.

Overall, the lack of significant increase in 25(OH)D levels in those who received the supplements could be a true finding because a meta-analysis of 76 trials of the influence of variable doses of vitamin D supplementation on serum 25-hydroxyvitamin D levels in Caucasians but not Asians reported that trials that used similar supplement doses could obtain significantly different changes to 25(OH)D concentrations [10].

Our most interesting finding is the decreased PTH concentration and increased calcium/creatinine ratio in subjects who received vitamin D and Calcium combination compared to those who received vitamin D alone or calcium alone. First, the high PTH does point to a degree of secondary hyperparathyroidism as a result of increased bone turnover associated with 25(OH)D deficiency. High PTH increase bone turnover and may also exert inhibitory effects on bone growth by increasing the production however, the relationship between PTH and 25(OH)D is known to be non-linear and more importantly the threshold of 25(OH)D level in relation to bone turnover and other health outcomes for Asian population is not definitely known [23]. Overall the health Implications of low vitamin D status in dark-skinned people are not clearly defined. Indeed, many studies on the relationship between low vitamin D status and bone health in south Asian population have yielded conflicting results. For example, a cross-sectional study evaluated whether Pakistanis living in the city of Oslo, have increased bone turnover compared with ethnic Norwegians due to their high prevalence of vitamin D deficiency. The results revealed only minor ethnic differences in bone turnover, despite a striking difference in prevalence of secondary hyperparathyroidism with no differences observed in bone mineral density between the two groups [6]. Another study from the UK on vitamin D status and markers of bone turnover in Caucasian and South Asian postmenopausal women found that although South Asian women had significantly higher serum parathyroid hormones (PTH) and lower 25(OH)D concentrations there was no significant differences between the two groups for biochemical markers of bone turnover [7]. Emerging evidence is also suggesting that increasing or adequate calcium intake by diet or use of supplement may mitigate the deleterious effects of low vitamin D concentrations on bone health by preventing or reducing bone turnover especially in darker-skinned people [7–9]. The minimum amount of dietary calcium required to induce this response is also not clear and may vary with ethnicity [7]. Again, very few studies have attempted to measure calcium intake in relation to bone health in areas where vitamin D deficiency is prevalent [7, 9–11]. Research is also required to examine the threshold of vitamin D and calcium intake and/or level in relation to bone turnover and other health outcomes in the UAE population. Overall our results indicate that the combination of vitamin D and calcium may mitigate the deleterious effects of vitamin D deficiency.

Another important area of enormous public health implication for our population is the association between low 25(OH)D levels and obesity and increased inflammation. This is because obesity and related diabetes is reaching epidemic proportions in the UAE and other Gulf countries and the UAE has one of the highest prevalence in the World [24, 25]. We have previously reported that abdominal obesity is more common in our population and associated with increased inflammation and decreased antioxidant status [26].Although obesity did not affect response to supplements in this study several observational studies have raised the possibility of the role of vitamin D in the development of type 2 diabetes and that low plasma 25(OH)D concentrations might be a mediator between obesity and increased risk of diabetes [27]. Experimental studies suggest 25(OH)D deficiency impairs glucose-induced insulin secretion and that insulin sensitivity may improve with 25(OH)D supplementation in patients with vitamin D deficiency [27–29]. Although, hitherto studies of the effects of vitamin D supplementations on obesity associated inflammation and risk of type 2 Diabetes have produced conflicting results [30–32], nevertheless, ongoing trial results should provide us with more definitive answers. This is because our population and similar populations from around the World have the most to gain from the result of these ongoing studies because of the co-existence of pathologically high proportions of obesity and vitamin D deficiency rates.

### Strength and limitations

Almost half of randomised subjects didn’t attend or refused to give a blood sample at the 6 months follow up. Another potential limitation is the frequency and dose of vitamin D supplement used in our study population of high rates of obesity and poor vitamin D status. Thirdly, adherence to supplements and possible overreporting of the number of tables taken in the light of the lack of expected increase in vitamin 25(OH)D levels in relation to supplements dose used and duration. Although compliance was assessed meticulously by counting tablets at follow up appointments in all study participants.

Nevertheless, the current study is, to our knowledge, the first randomized, double-blind, placebo-controlled trial to investigate the effect of vitamin D supplementation with or without calcium on metabolic and bone turnover markers and general health in community-dwelling vitamin D deficient UAE citizens. Despite the high number of participants lost to follow up and modest compliance with the full amounts of prescribed supplements we compared the group randomly allocated to supplements with the group allocated to placebo. This approach, in addition to being unbiased, does provide a pragmatic answer to the question of primary clinical interest in this trial – e.g., does vitamin D3 and calcium supplementation, either alone or in combination, have health benefit? In addition, based on pre-study sample size calculation the number of participants with follow up data is large enough to detect a difference if one exists and compliance was assessed meticulously by counting tablets at follow up appointments in all study participants.

## Conclusion

Although we found no evidence of a clinical benefit of vitamin D supplementation with or without calcium, we found decreased PTH concentration and increased calcium/creatinine ratio in subjects who received vitamin D and Calcium combination. Increasing or adequate calcium intake in combination with vitamin D may mitigate the deleterious health effects of low vitamin D concentrations, however more research is required to examine this preliminary finding in areas where vitamin D deficiency is prevalent. Research is also needed to test emerging evidence that increased dietary intake or oral supplementation with calcium may mitigate the deleterious health effects of low vitamin D concentrations. There is also an urgent need to study the effects of higher dose, frequency and duration of vitamin D supplementation including more testing and adherence to the supplements to reach and sustain higher levels of 25(OH)D particularly in high-risk populations.

## Data Availability

The data set are not publicly available but are available from the corresponding author on reasonable request.
